# Chronic Ethanol Consumption Disrupts the Core Molecular Clock and Diurnal Rhythms of Metabolic Genes in the Liver without Affecting the Suprachiasmatic Nucleus

**DOI:** 10.1371/journal.pone.0071684

**Published:** 2013-08-12

**Authors:** Ashley N. Filiano, Telisha Millender-Swain, Russell Johnson, Martin E. Young, Karen L. Gamble, Shannon M. Bailey

**Affiliations:** 1 Department of Pathology-Division of Molecular and Cellular Pathology, University of Alabama at Birmingham, Birmingham, Alabama, United States of America; 2 Department of Psychiatry-Division of Behavioral Neurobiology, University of Alabama at Birmingham, Birmingham, Alabama, United States of America; 3 Department of Medicine-Division of Cardiovascular Diseases, University of Alabama at Birmingham, Birmingham, Alabama, United States of America; University of Massachusetts Medical School, United States of America

## Abstract

Chronic ethanol consumption disrupts several metabolic pathways including β-oxidation and lipid biosynthesis, facilitating the development of alcoholic fatty liver disease. Many of these same metabolic pathways are directly regulated by cell autonomous circadian clocks, and recent studies suggest that disruption of daily rhythms in metabolism contributes to multiple common cardiometabolic diseases (including non-alcoholic fatty liver disease). However, it is not known whether ethanol disrupts the core molecular clock in the liver, nor whether this, in turn, alters rhythms in lipid metabolism. Herein, we tested the hypothesis that chronic ethanol consumption disrupts the molecular circadian clock in the liver and potentially changes the diurnal expression patterns of lipid metabolism genes. Consistent with previous studies, male C57BL/6J mice fed an ethanol-containing diet exhibited higher levels of liver triglycerides compared to control mice, indicating hepatic steatosis. Further, the diurnal oscillations of core clock genes (*Bmal1*, *Clock*, *Cry1*, *Cry2*, *Per1*, and *Per2*) and clock-controlled genes (*Dbp*, *Hlf*, *Nocturnin*, *Npas2*, *Rev*-*erbα*, and *Tef*) were altered in livers from ethanol-fed mice. In contrast, ethanol had only minor effects on the expression of core clock genes in the suprachiasmatic nucleus (SCN). These results were confirmed in *Per2^Luciferase^* knock-in mice, in which ethanol induced a phase advance in PER2::LUC bioluminescence oscillations in liver, but not SCN. Further, there was greater variability in the phase of PER2::LUC oscillations in livers from ethanol-fed mice. Ethanol consumption also affected the diurnal oscillations of metabolic genes, including *Adh1*, *Cpt1a*, *Cyp2e1*, *Pck1, Pdk4, Ppargc1a*, *Ppargc1b* and *Srebp1c*, in the livers of C57BL/6J mice. In summary, chronic ethanol consumption alters the function of the circadian clock in liver. Importantly, these results suggest that chronic ethanol consumption, at levels sufficient to cause steatosis, disrupts the core hepatic clock as well as the diurnal rhythms of key lipid metabolism genes**.**

## Introduction

Chronic alcohol consumption is a significant public health problem with approximately 12,000 deaths per year attributed to alcoholic liver disease [1]. Liver disease from chronic alcohol consumption is a spectrum of disorders ranging from simple steatosis to steatohepatitis and cirrhosis. More than 90% of heavy drinkers develop steatosis, accumulation of lipid droplets largely comprised of triglycerides, phospholipids, and cholesterol esters, within hepatocytes [2]. This ectopic lipid accumulation is thought to result from alcohol-induced stimulation of lipogenesis and inhibition of fatty acid oxidation [2], [3]. In particular, alcohol increases the levels of active sterol regulatory element binding protein 1c (SREBP-1c), a transcription factor that regulates genes involved in lipogenesis, including fatty acid synthase (*FASN*) and acetyl-CoA carboxylase (*ACC*) [4]. These effects on SREBP-1c protein could be an indirect result of alcohol-induced inhibition of AMP-activated protein kinase (AMPK) or sirtuin-1 (SIRT1); two proteins that reduce SREBP-1c activity [5], [6]. Further, acetaldehyde, a metabolite of ethanol, directly activates SREBP-1c [4]. On the other hand, acetaldehyde inhibits the transactivation and DNA binding activities of peroxisome proliferator-activated receptor alpha (PPARα), a nuclear hormone receptor that controls genes involved in fatty acid transport and oxidation, such as carnitine palmitoyl transferase 1 (*CPT1*), fatty acid binding protein (*FABP*), and acyl CoA dehydrogenase (*ACADM*) [3]. This ethanol-induced increase in lipid biosynthesis, combined with a decrease in fatty acid oxidation, likely contributes to the development of fatty liver.

Within the past decade, it has been shown that many physiological functions, including lipid metabolism, glucose homeostasis, and insulin sensitivity, are regulated by molecular circadian clocks [7]–[10]. Peripheral clocks, located in virtually all tissues in the body, are synchronized to external cues, called Zeitgebers (“time givers”), through neural and humoral signals received from the master clock within the suprachiasmatic nucleus (SCN) of the hypothalamus [11]. Peripheral clocks can also be regulated independently of the SCN by other cues such as food [12], [13]. These clocks are molecular oscillators consisting of transcriptional-translational feedback loops. On the positive side of this loop is a heterodimer consisting of the proteins circadian locomotor output cycles kaput (CLOCK) and Aryl hydrocarbon receptor nuclear translocator-like (ARNTL/BMAL1) that binds E-box elements in the promoters of other clock genes, including period 1, 2, and 3 (*PER1*, *PER2*, *PER3*), cryptochrome 1 and 2 (*CRY1*, *CRY2*), retinoic acid related orphan receptor alpha (*RORA*), and nuclear receptor subfamily 1, group D, member 1 (*NR1D1*/*REVERBα*). The PER and CRY proteins, which comprise the negative side of the loop, heterodimerize with one another and inhibit the CLOCK:BMAL1 complex [14]. RORA and REVERBα form a second feedback loop by directly influencing the transcription of *BMAL1* [14]. These clock oscillators aid in confining physiological pathways to metabolically necessary times of the day through transcriptional and post-translational regulation of multiple metabolic pathways, including those influencing lipid metabolism.

Alterations in circadian clocks via genetic circadian disruption [15] or circadian desynchrony [16] (defined as modified phase relationships among internal biological rhythms or between internal rhythms and the external environment) are associated with the development of a number of diseases. These include, but are not limited to, cancer [17], metabolic syndrome [9], [15], diabetes [18] and cardiovascular disease [19]. For example, *Bmal1* knockout mice fed a high fat diet develop symptoms similar to metabolic syndrome, including ectopic accumulation of fat within the liver [9]. Further, chronic ethanol consumption alters expression of lipid metabolism genes in the livers of *Clock*-mutant mice [20] and disrupts circadian activity rhythms in wild-type mice [21]. Based on this, we hypothesize that chronic ethanol consumption alters the liver molecular circadian clock and potentially affects the diurnal rhythms of lipid metabolism genes in the liver. In the present study, we tested this hypothesis by feeding male mice a control or ethanol-containing liquid diet and examining liver triglyceride content and expression of clock and metabolic genes in the liver at multiple times during a 24-hr period. Clock gene expression was also measured in the SCN. We also examined PER2::LUC bioluminescence activity in *ex vivo* liver and SCN tissue explants from *Per2^Luciferase(Luc)^* mice fed a control or ethanol-containing diet under the same conditions. Results from this study suggest that chronic ethanol consumption disrupts the peripheral clock in the liver as well as diurnal rhythms of key lipid metabolism genes that have been previously associated with development of alcoholic fatty liver disease.

## Materials and Methods

### Ethics Statement

All animal procedures were approved by the Institutional Animal Care and Use Committee at the University of Alabama at Birmingham (animal project number 9178) and complied with NIH guidelines.

### Diet Formulation

Mice were maintained on a normal chow diet (NIH-31 Open Formula Mouse/Rat Sterilizable Diet 7917, Teklad Diets, Madison, Wisconsin) before beginning the ethanol feeding study. Lieber-DeCarli control and ethanol liquid diets were purchased from Bio-Serv (Frenchtown, New Jersey, USA) and were formulated as described in [22]. The nutrient profile of each diet was identical, excluding carbohydrate content. Ethanol and control diets both contained 15.1% calories from protein and 35.9% calories from fat. Control diet contained 49% calories from carbohydrates, while there were 20.6% calories from carbohydrates in the ethanol diet. The remaining 28.4% of calories in the ethanol diet was from ethanol.

### Mice and Ethanol Feeding Procedure

Male C57BL/6J (Jackson Labs, Bar Harbor, Maine) and *Per2^Luc^* heterozygous mice were 8 weeks old at the beginning of the feeding studies. The *Per2^Luc^* mouse colony (maintained by Dr. Karen Gamble, UAB) was founded from *Per2^Luc^* homozygous mice [23] (Jackson Labs) crossed with wild-type C57BL/6J to obtain heterozygous *Per2^Luc^* mice for experiments. This mouse line was crossed for 3 generations prior to experimentation. *Per2^Luc^* mice contain the *Luc* gene fused in-frame to the 3′ end of the endogenous *Per2* gene. This system serves as a real-time reporter of PER2::LUC expression in all tissues.

Mice were weight matched and pair-fed the Lieber-DeCarli diet with or without ethanol. Mice were fed daily at ZT 9–11. The control mouse in each pair was provided with the amount of food that its ethanol partner consumed the previous night, so that each pair was isocaloric at the end of the feeding period. *Per2^Luc^* and C57BL/6J mice were maintained on the diets for 35–37 and 29–36 days, respectively, before they were used in experiments. Mice were single-housed at 22–23°C under a 12∶12 hr light-dark cycle, allowing us to measure diurnal changes in gene expression and liver triglyceride content. Light intensity at cage level was 40- 50 lux. Mice were weighed weekly and had unlimited access to water.

### Tissue Collection and Biochemical Measurements

Tissues were collected at Zeitgeber Time (ZT) 3, 7, 11, 15, 19, and 23 (where ZT 0 = lights on and ZT 12 = lights off), unless otherwise noted. At ZT 15, 19, and 23, an infrared viewer was utilized for tissue collection and photic input to the SCN was blocked prior to exposure to room light by removing eyes. Brain sections (600 µm) were prepared on a vibroslicer (Campden 7000SMZ; World Precision Instruments, Lafayette, IN) in cold Hank’s Balanced Salt Solution (Life Technologies, Grand Island, NY) and the SCN was carefully dissected under a dissection microscope [24]. Liver sections from *Per2^Luc^* mice were dissected from the outer area of the large lobe. SCN from C57BL6/J mice and liver pieces were snap frozen in liquid nitrogen for biochemical or gene expression analysis. Whole blood was collected into BD Microtainer Tubes with EDTA (Becton, Dickinson, and Company, Franklin Lakes, NJ) for plasma separation. Blood alcohol levels were determined using the Alcohol Reagent Set (Pointe Scientific, Inc., Canton, Michigan). Plasma alanine aminotransferase (ALT) levels were determined using the ALT (SGPT) Reagent Set (Point Scientific, Inc., Canton, Michigan).

### *Ex Vivo* Tissue Culture

Liver and SCN from *Per2^Luc^* mice [23] were harvested between ZT 3–5. Coronal sections (250 µm) were made using a vibroslicer with cold sampling media [24], [25]. SCN sections were transferred to Millicell culture plate inserts (Millipore, Ballerica, MA) in 35 mm cell culture dishes containing 1.0 mL of recording medium [24], [25] and circular cover glass lids (VWR International, Radnor, PA) were sealed with silicon grease. Liver sections from each animal were dissected from the outer area of the large lobe under a dissection microscope, rinsed with cold sampling media, and transferred to a mesh macroporous filter (Spectrum Laboratories, Inc., Rancho Dominguez, CA). Each liver section was placed separately into a 35 mm cell culture dish containing 1.5 mL of recording media supplemented with putrescine (0.1 mM; MP Biomedicals, LLC, Solon, OH) and insulin (10 ng/mL; Roche). Culture dishes were placed in a Lumicycle luminometer (Actimetrics, Wilmette, IL) and luminescence was measured every 6 min. Phase was defined as the time of the peak of the first full cycle using Lumicycle data analysis software (Actimetrics). For the first four cycles, the baseline shift was removed by fitting a polynomial curve with an order of the number of cycles or one less than the number of cycles. For each mouse, the liver culture with the best goodness of fit, accounting for at least 80% of the variance in the time-series data was used for analysis. SCN sections from every animal met this same criterion and were included in data analysis.

### Lipid Extraction and Triglyceride Measurement

Total lipids were extracted from liver using a modified Folch procedure [26]. Approximately 10 mg of liver tissue was placed in 5 mL of a 2∶1 solution of chloroform and methanol and homogenized using a Polytron homogenizer (PT1200E, Kinematica, Bohemia, NY). Samples were agitated for 2 hr at room temperature and tissue was filtered. Liquid phases were separated through addition of 1 mL 0.05% sulfuric acid and the bottom phase was obtained. Prior to triglyceride determination, 4 mL of 1% Triton X-100 in chloroform was added and chloroform was evaporated under nitrogen gas. Lipids were dissolved in deionized water. Triglyceride content was determined in triplicate in a 96 well plate using the L-Type TG M colorimetric assay (Wako Diagnostics, Osaka, Japan). Absorbance at 595 nm was determined using a Multiscan Spectrum Microplate Reader (Thermo Scientific).

### RNA Isolation and Gene Expression Analysis

Total RNA was isolated from liver and SCN using TriReagent (Sigma, St. Louis, MO) following manufacturer’s instructions. Samples were DNase treated using the DNA-free™ kit (Invitrogen, Grand Island, NY). Reverse transcription was performed using the High Capacity cDNA Reverse Transcription kit (Applied Biosystems, Carlsbad, CA). Real-time PCR was performed using an Applied Biosystems 7900 HT instrument. Taqman Gene Expression assays containing gene specific primers and probes were also purchased from Applied Biosystems. The complete list of genes examined in liver and SCN is included in Table S1. Relative gene expression was determined using the comparative cycle threshold method. Expression was normalized to the *Gapdh* housekeeping gene and represented as fold-change from a single control mouse.

### Statistics

Data are presented as mean ± standard error of the mean for at least n = 4 control or ethanol-fed mice, unless otherwise noted in figure legends. Two-way repeated measures ANOVA was utilized to determine differences in total body weight between control and ethanol-fed mice. Significant differences in blood alcohol levels in C57BL/6J mice were determined using One-way ANOVA. Significant differences in liver/body weight ratios and liver triglycerides and SCN-liver phase angles of PER2::LUC mice were determined using a t-test. The non-parametric Mann-Whitney Rank Sum Test was utilized to assess statistical differences in plasma ALT levels as data were not normally distributed. Two-way ANOVA was used to determine significant time and/or treatment effects on gene expression and liver triglyceride content in C57BL/6J mice. Cosinor analysis [27], [28] using the Nonlinear Regression module within SPSS was used to fit a cosine curve to gene expression data that demonstrated significant time effects according to Two-way ANOVA. Cosinor analysis utilizes the following equation: f(t) = Mesor+A * cos[(2πt/T)+ Acrophase] with the Mesor (midline estimating statistic of rhythm) = mean of the oscillation; A = amplitude (1/2 the distance between the peak and the trough); t = a timepoint (ZT 3, 7, 11, 15, 19, or 23); T = the period (manually set to 24 hr for the analysis); and Acrophase = the ZT time of the cosine maximum. An r-squared value was given for each fitted cosine curve to determine the percent variance accounted for by a 24 hr rhythm. Mesor, amplitude, and acrophase estimates from the nonlinear regression model for each treatment group (control vs. ethanol) were compared using t-tests. Rayleigh plots showing phases of PER2::LUC expression in SCN and liver were generated using Oriana software and significant phase differences were determined using a Watson-Williams F-Test. The level of significance for all tests was set at *p*<0.05. Statistical analyses were performed in SPSS, Sigma Plot, and Microsoft Excel.

## Results

### C57BL/6J Mice and Liver Histology

**Figure 1:**
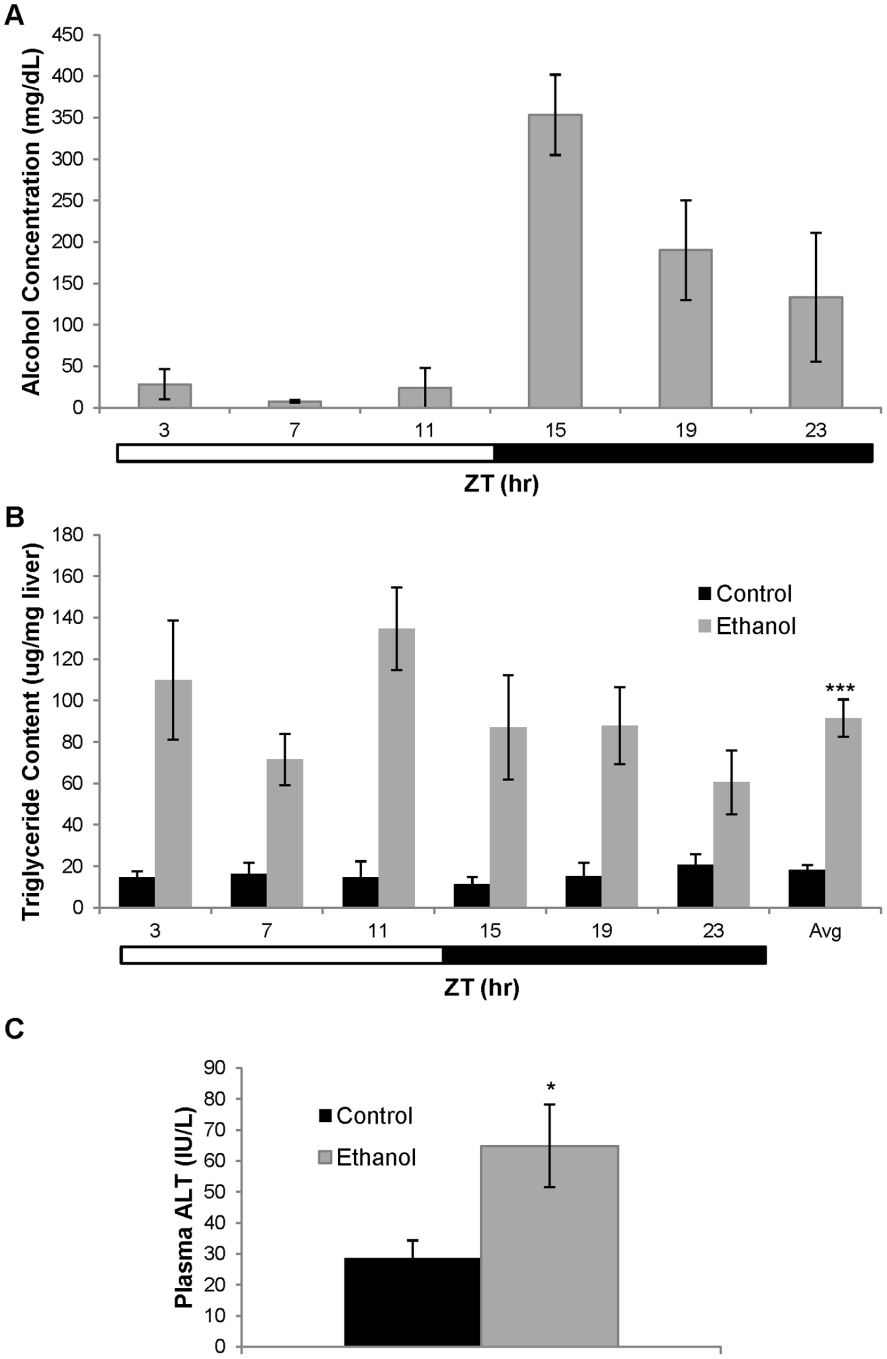
Biochemical measurements in C57BL/6J mice. A) Blood alcohol concentration (BAC) in ethanol-fed C57BL/6J mice. One-way ANOVA analysis revealed significant changes in BAC over a 24 hour period (p<0.001). Data are presented as mean ± SEM for 3–5 ethanol-fed mice at each ZT time. B) Triglyceride content in livers from control (▪) or ethanol-fed (grey square) mice. Two-way ANOVA was performed to determine significant effects of time and/or treatment on triglyceride content. There was no main effect of time (ZT) on liver triglyceride levels (*F*(5,52) = 1.595, p = 0.178) and no significant interaction between ZT time and diet (*F*(5,52) = 1.995, *p = *0.095). However, there was a significant main effect of diet (*F*(1,52) = 91.125, p<0.0001). Significant differences in mean triglyceride content across all ZT times were determined using a t-test. Data are presented as mean±SEM for 4–6 control and ethanol-fed mice at each time point with the mean triglyceride content across all times shown in the last set of bars (n = 28 control and 28 ethanol-fed mice). ***p<0.0001, compared to control. C) Plasma ALT levels in control (▪) and ethanol-fed (grey square) mice. ALT levels were significantly increased in ethanol-fed mice as determined by Mann-Whitney Rank Sum Test (*p = 0.019). Data are presented as mean ± SEM for 21 control mice and 16 ethanol-fed mice.

Wild-type C57BL/6J mice were pair-fed a control or an ethanol-containing liquid diet for at least 4 wk. Body weights were monitored over the course of the feeding protocol (Figure S1a). There was no significant main effect of diet on body weight (*F(*1,256) = 0.170, *p = *0.682) as determined by a two-way repeated measures ANOVA; however, there was a significant main effect of time (*F*(4,256) = 10.775, *p*<0.001). At the end of the feeding protocol, liver, SCN, and blood were collected at 4-hr intervals for a period of 24-hr (ZT 3, 7, 11, 15, 19, and 23). Blood alcohol concentration (BAC) was measured in ethanol-fed animals (Figure 1a). As expected, there was a significant effect of time on BAC as determined by one-way ANOVA (p<0.001), with peak blood alcohol levels occurring at ZT 15 (353.3±48.5 mg/dL) followed by a decrease in BAC extending into the normal inactive period reaching its lowest point at ZT 7 (7.6±1.6 mg/dL).

Liver parameters were also measured in both groups. There was a slight, yet significant, increase in liver to body weight ratio in mice fed an ethanol-containing diet compared to controls (4.68±0.11% versus 4.12±0.11%, *p* = 0.0003). There was no main effect of ZT time on liver triglyceride levels as determined by two-way ANOVA (*F*(5,52) = 1.595, *p = *0.178); however, there was a significant main effect of diet, (*F*(1,52) = 91.125, p<0.0001). Specifically, liver triglyceride levels were significantly higher (p<0.0001) in ethanol-fed animals (91.5±9.1 µg/mg liver; n = 28) when compared to controls (18.2±2.4 µg/mg liver; n = 28) (Figure 1b). There was no significant interaction between ZT time and diet (*F*(5,52) = 1.995, *p = *0.095). Further, there was a significant increase (p<0.05) in ALT levels in ethanol-fed animals compared to controls (64.9±13.3 versus 28.7±5.64 IU/L) (Figure 1c). These data collectively indicate that our ethanol feeding protocol induced steatosis, the early stage of alcoholic liver disease.

### Chronic Ethanol Consumption Affects Diurnal Variations in Liver Clock Genes

**Figure 2:**
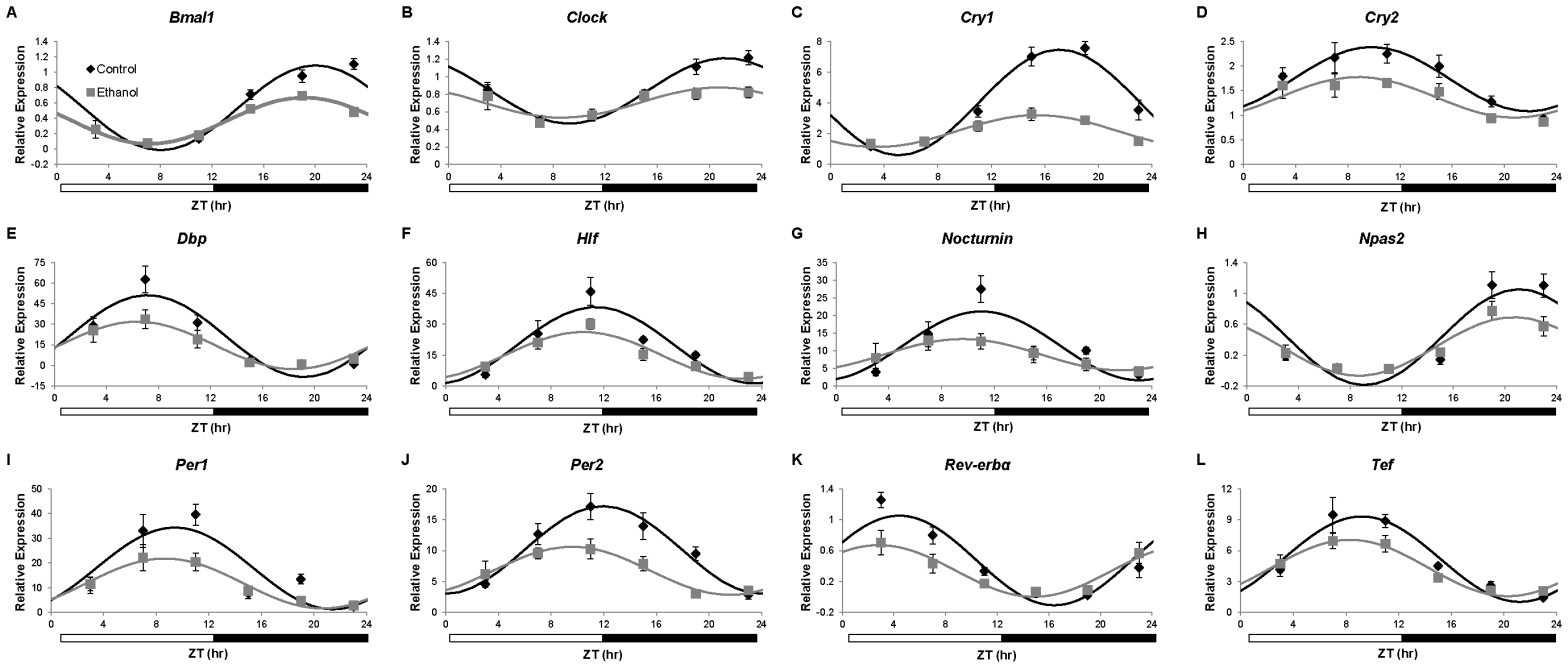
Expression of clock genes in the liver of C57BL/6J mice. Gene expression of A) *Bmal1*, B) *Clock*, C) *Cry1*, D) *Cry2*, E) *Dbp*, F) *Hlf*, G) *Nocturnin*, H) *Npas2*, I) *Per1*, J) *Per2*, K) *Rev-erbα*, and L) *Tef* in liver from mice fed control (♦) or ethanol-containing (grey square) diets was determined using real-time quantitative PCR. Expression levels were normalized to the *Gapdh* housekeeping gene and are displayed as fold-change from a single control mouse. Cosinor analysis for curve fitting was performed using the nonlinear regression module in SPSS. Cosinor analysis results are included in Table 1. Data are presented as mean ± SEM for n = 4–6 control and ethanol-fed mice at each time point.

To determine if ethanol affects the circadian clock in the liver, mRNA expression of *Bmal1*, *Clock*, *Cry1*, *Cry2*, *Dbp*, *Hlf*, *Nocturnin*, *Npas2*, *Per1*, *Per2*, *Rev*-*erbα*, and *Tef* was measured in livers collected at multiple times. There was a significant main effect of ZT time on liver expression of all twelve genes examined, as determined by two-way ANOVA (Table S2). Further, cosinor analysis revealed significant diurnal oscillations in hepatic expression of all clock genes in both control and ethanol-fed mice (Table 1, Figure 2). Ethanol significantly decreased the mesor of several core clock genes (*Bmal1*, *Clock*, *Cry1*, *Cry2*, *Per1*, and *Per2*), as well as clock-controlled genes (*Dbp*, *Hlf*, and *Rev*-*erbα*). The amplitude (1/2 the distance between peak and trough) of *Bmal1*, *Clock*, *Cry1*, *Dbp*, *Hlf*, *Nocturnin*, *Npas2*, *Per2*, *Rev*-*erbα*, and *Tef* diurnal oscillations were also significantly reduced in the livers of mice fed the ethanol-containing diet. Interestingly, ethanol induced a phase advance (peak expression occurred earlier) in three genes examined. Specifically, *in vivo* liver expression levels of *Cry1*, *Per2*, and *Rev*-*erbα* were phase advanced by 1.63, 2.38, and 1.58 hr, respectively, in ethanol-fed mice. These results demonstrate that chronic ethanol consumption alters the peripheral clock within the liver.

**Table 1:** Cosinor analysis of clock genes in the liver.

Gene		r^2^	p-value	Mesor	p-value	Amplitude	p-value	Acrophase	p-value
*Bmal1*	Control	0.85	<0.0001	0.54	0.0001	0.55	<0.0001	20.04	0.0793
	Ethanol	0.78	<0.0001	0.37		0.30		19.15	
*Clock*	Control	0.772	<0.0001	0.84	0.0040	0.37	0.0025	21.23	0.6227
	Ethanol	0.31	0.0239	0.70		0.17		20.68	
*Cry1*	Control	0.83	<0.0001	4.03	<0.0001	3.44	<0.0001	17.08	.01090
	Ethanol	0.68	<0.0001	2.17		1.04		15.45	
*Cry2*	Control	0.508	<0.0001	1.73	0.0017	0.65	0.1292	9.82	0.3597
	Ethanol	0.385	0.0062	1.36		0.41		8.78	
*Dbp*	Control	0.695	<0.0001	21.33	0.0447	29.88	0.0099	7.16	0.3335
	Ethanol	0.584	0.0001	14.51		17.35		6.39	
*Hlf*	Control	0.626	<0.0001	19.75	0.0242	18.57	0.0220	11.39	0.1773
	Ethanol	0.714	<0.0001	14.80		11.40		10.44	
*Nocturnin*	Control	0.495	<0.0001	11.34	0.1260	9.74	0.0247	11.04	0.2804
	Ethanol	0.28	0.0392	8.86		4.43		9.61	
*Npas2*	Control	0.696	<0.0001	0.43	0.0601	0.62	0.0110	21.10	0.5140
	Ethanol	0.693	<0.0001	0.31		0.38		20.67	
*Per1*	Control	0.54	<0.0001	17.78	0.0117	16.53	0.0575	9.42	0.3931
	Ethanol	0.59	<0.0001	11.66		10.14		8.66	
*Per2*	Control	0.67	<0.0001	10.10	0.0002	7.10	0.0087	11.96	0.0051
	Ethanol	0.55	0.0002	6.72		3.91		9.58	
*Rev-erb*α	Control	0.752	<0.0001	0.47	0.0248	0.58	0.0048	4.42	0.0419
	Ethanol	0.584	0.0001	0.34		0.33		2.84	
*Tef*	Control	0.69	<0.0001	5.17	0.0524	4.14	0.0268	9.20	0.1677
	Ethanol	0.718	<0.0001	4.31		2.73		8.28	

### Chronic Ethanol Consumption Does Not Affect the Mesor or Amplitude of Diurnal Variations in Clock Genes in the SCN

**Figure 3:**
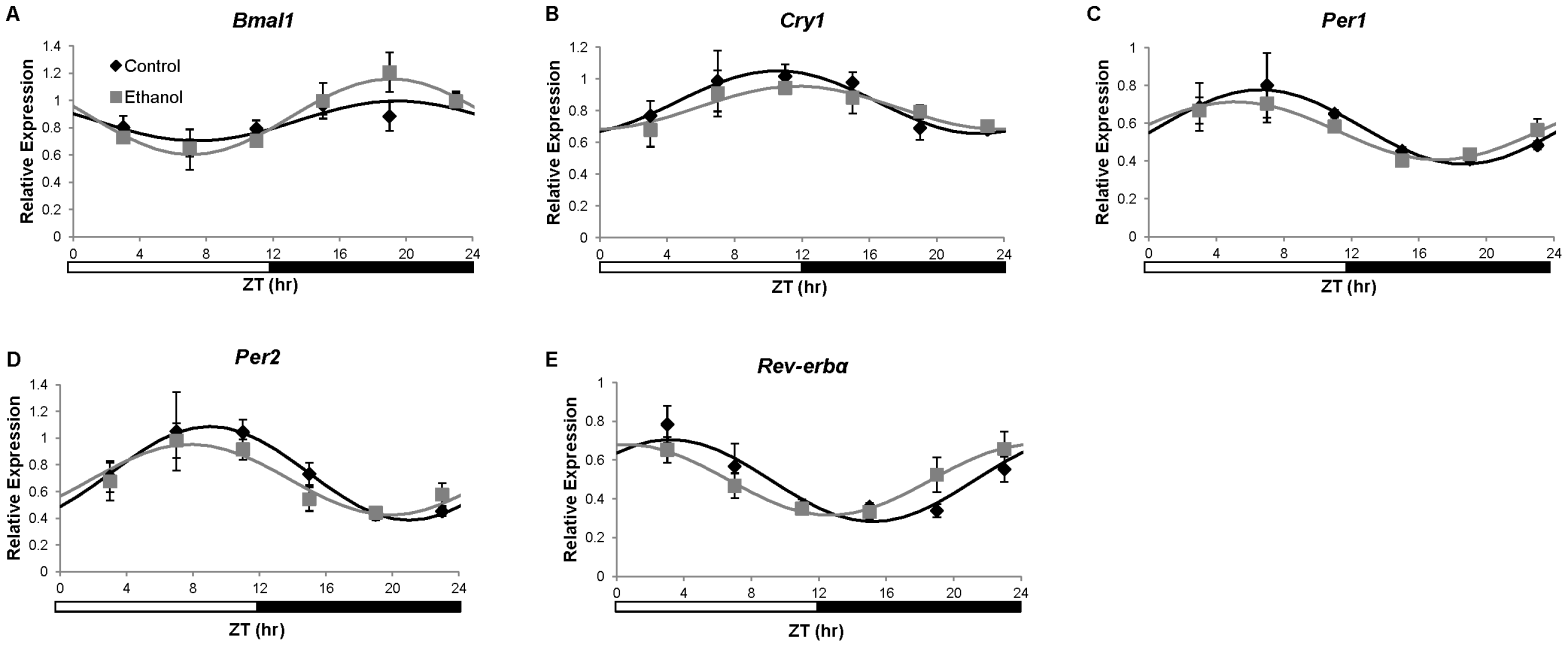
Expression of clock genes in the suprachiasmatic nucleus (SCN) of C57BL/6J mice. Gene expression of a) *Bmal1*, b) *Cry1*, c) *Per1*, d) *Per2*, and e) *Rev-erbα* in SCN from mice fed control (♦) or ethanol-containing (grey square) liquid diets was determined using real-time quantitative PCR. Expression levels were normalized to the *Gapdh* housekeeping gene and are displayed as fold-change from a single control mouse. Cosinor analysis for curve fitting was performed using the nonlinear regression module in SPSS. Cosinor analysis results are included in Table 2. There were no statistically significant differences between the two feeding groups in mesor or amplitude in any genes examined in the SCN. Data are presented as mean ± SEM for n = 4 control and ethanol-fed mice at each time point.

To determine if the ethanol-dependent alterations in the liver clock were due to dysregulation of the circadian clock in the SCN, we examined mRNA expression of *Bmal1*, *Clock*, *Cry1*, *Cry2*, *Per1*, *Per2*, *Rev*-*erbα*, and *Tef* in SCN samples collected at multiple times during a 24 hr period. There was no significant main effect of ethanol feeding on expression of SCN clock genes examined as determined by two-way ANOVA (Table S3). Further, there was no significant main effect of time on the expression of *Clock*, *Cry2*, or *Tef* and there were no significant interactions. ZT time did have a significant main effect on the expression of *Bmal1*, *Cry1*, *Per1*, *Per2*, and *Rev*-*erbα* (Table S3). The time-dependent variations of *Cry1*, *Per1*, *Per2*, and *Rev*-*erbα* in control mice were significantly diurnally regulated as determined by cosinor analysis (Table 2, Figure 3). *Bmal1* expression did not fit a cosine function in control mice (p = 0.0921). Significant diurnal oscillations in SCN expression of *Bmal1*, *Per1*, *Per2*, and *Rev*-*erbα,* but not *Cry1*, were shown in ethanol-fed mice. Ethanol had no significant effect on the amplitude, acrophase, or mesor of *Per1* or *Per2*. Further, ethanol did not affect the acrophase or mesor of *Rev*-*erbα*, but did induce a 2.55 hr phase advance in SCN expression of this gene (Table 2, Figure 3e). Overall, the effects of ethanol on SCN clock gene expression were not as striking as those observed in the livers of ethanol-fed mice, suggesting that the effects of ethanol on the liver clock are not a downstream effect of overt central clock alteration.

**Table 2:** Cosinor analysis of clock genes in the SCN.

Gene		r^2^	p-value	Mesor	p-value	Amplitude	p-value	Acrophase	p-value
*Bmal1*	Control	0.26	0.0921	**–**	**–**	**–**	**–**	**–**	**–**
	Ethanol	0.61	0.0002	**–**		**–**		**–**	
*Cry1*	Control	0.38	0.0153	**–**	**–**	**–**	**–**	**–**	**–**
	Ethanol	0.27	0.0758	**–**		**–**		**–**	
*Per1*	Control	0.45	0.0049	0.58	0.6134	0.20	0.4584	6.63	0.2390
	Ethanol	0.53	0.0010	0.56		0.15		5.15	
*Per2*	Control	0.51	0.0016	0.74	0.4850	0.35	0.3673	9.01	0.3227
	Ethanol	0.52	0.0012	0.69		0.27		7.85	
*Rev-erb*α	Control	0.56	0.0007	0.49	0.9293	0.21	0.5926	3.19	0.0240
	Ethanol	0.55	0.0008	0.50		0.18		0.64	

### Per2^Luc^ Mice and Liver Triglycerides

We showed that chronic ethanol consumption affects the clock in the liver without dramatically affecting the core clock components in the SCN. We next sought to confirm these studies using a mouse model in which clock gene expression is assessed in real-time within a single mouse. We utilized the *Per2^Luc^* mouse model, which allowed us to examine *ex vivo* luciferase activity as an output of *Per2* expression over an extended period of time in SCN and liver explants obtained from the same mouse. *Per2^Luc^* mice were pair-fed a control or ethanol-containing liquid diet. There was no significant main effect of diet on body weight over the course of the feeding protocol as determined by two-way repeated measures ANOVA (*F*(1,72) = 1.048, *p* = 0.32). However, there was a significant main effect of time (*F*(4,72) = 4.291, *p* = 0.004) as well as a significant interaction between time and diet (*F*(4,72) = 5.545, p<0.001) (Figure S1b). There was a significant increase in triglyceride content in the livers of ethanol-fed mice (117.6±11.2 µg/mg liver, n = 10) compared to control mice (49.7±6.9 µg/mg liver, n = 10), indicating ethanol-induced liver steatosis (p<0.0001).

### Chronic Alcohol Consumption Affects Circadian Expression of PER2::LUC in the Liver but not the SCN

**Figure 4:**
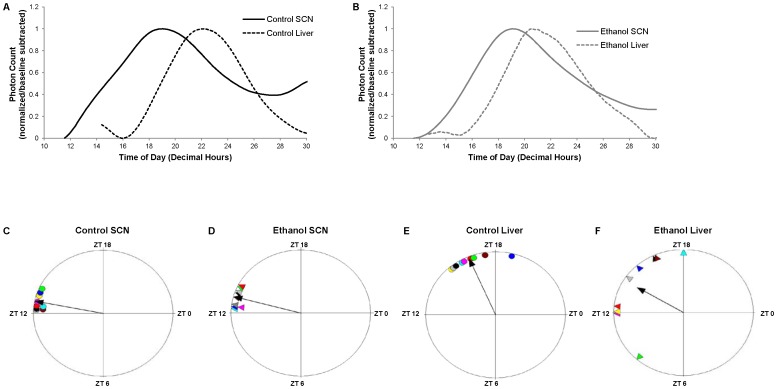
PER2::LUC expression in SCN and liver tissue explants. Representative traces of PER2::LUC bioluminescence in liver and SCN are shown for a pair-fed A) control and B) ethanol-fed mouse. Peak phases of PER2::LUC expression in C–D) SCN and E–F) liver tissue cultures from individual control (•) and ethanol-fed (▴) mice are shown on the first full cycle following culture. Corresponding colors between ethanol-fed and control mice indicate those that were pair-fed. Further, correspondingly colored shapes between SCN and liver indicate that those tissues were obtained from the same mouse. The black arrow indicates the mean phase vector of the samples, where length is inversely proportional to the phase variance, and the direction indicates timing relative to the previous light cycle in LD. The plots were generated using Oriana 3 software. Data represent results from n = 9 control and 9 ethanol-fed mice.

For these experiments, liver and SCN were harvested from *Per2^Luc^* mice at the beginning of the circadian day (ZT 3–5). Liver and SCN sections were cultured and PER2::LUC bioluminescence was measured. Representative traces of PER2::LUC bioluminescence in liver and SCN from a pair-fed control mouse (Figure 4a) and ethanol-fed mouse (Figure 4b) are provided. There was no difference in period length of PER2::LUC expression in liver or SCN sections between control and ethanol-fed mice. Figure 4c-f shows the phase of peak PER2::LUC expression in individual tissue cultures during the first full cycle after culture. The average time of the first peak of PER2::LUC bioluminescence in SCN occurred at ZT 12.75±0.17 and 13.00±0.20 in control and ethanol-fed mice, respectively (Figure 4c and 4d). This difference was not statistically significant (Watson-Williams F test, *F(*16,1) = 0.97, *p* = 0.339), demonstrating that chronic ethanol consumption had no effect on the phase of PER2::LUC in the SCN. The average phase of PER2::LUC expression occurred significantly earlier in liver sections from ethanol-fed mice as compared to controls (ZT 13.97±0.96 vs. ZT 16.47±0.39, respectively, (*F*(16,1) = 5.444, *p* = 0.033) (Figure 4e and 4f). These data are in agreement with our studies in wild-type C57BL/6 animals demonstrating that ethanol alters the clock within the liver without affecting the clock in the SCN.

The average SCN-liver phase angle was 0.96±1.05 hr in ethanol-fed mice and 3.72±0.36 hr in control mice. This difference was significant (paired t-test, *t*(8) = 2.85, p = 0.02), demonstrating an average phase advance of ∼2.8 hr in PER2::LUC liver expression in ethanol-fed mice. More importantly, there was a significantly larger variance in the SCN-liver phase angle of PER2::LUC in ethanol-fed mice compared to controls (Levene’s, p<0.05). SCN-liver phase angles in ethanol-fed mice ranged between −4.84 hr and 5.73 hr (Figure 4 d and f). In four mice, PER2::LUC expression peaked in the liver prior to the SCN. These data show that ethanol causes desynchrony between the peripheral clock in the liver and the master clock in the SCN.

### Chronic Alcohol Consumption Affects Expression of Metabolic Genes in the Liver

**Figure 5:**
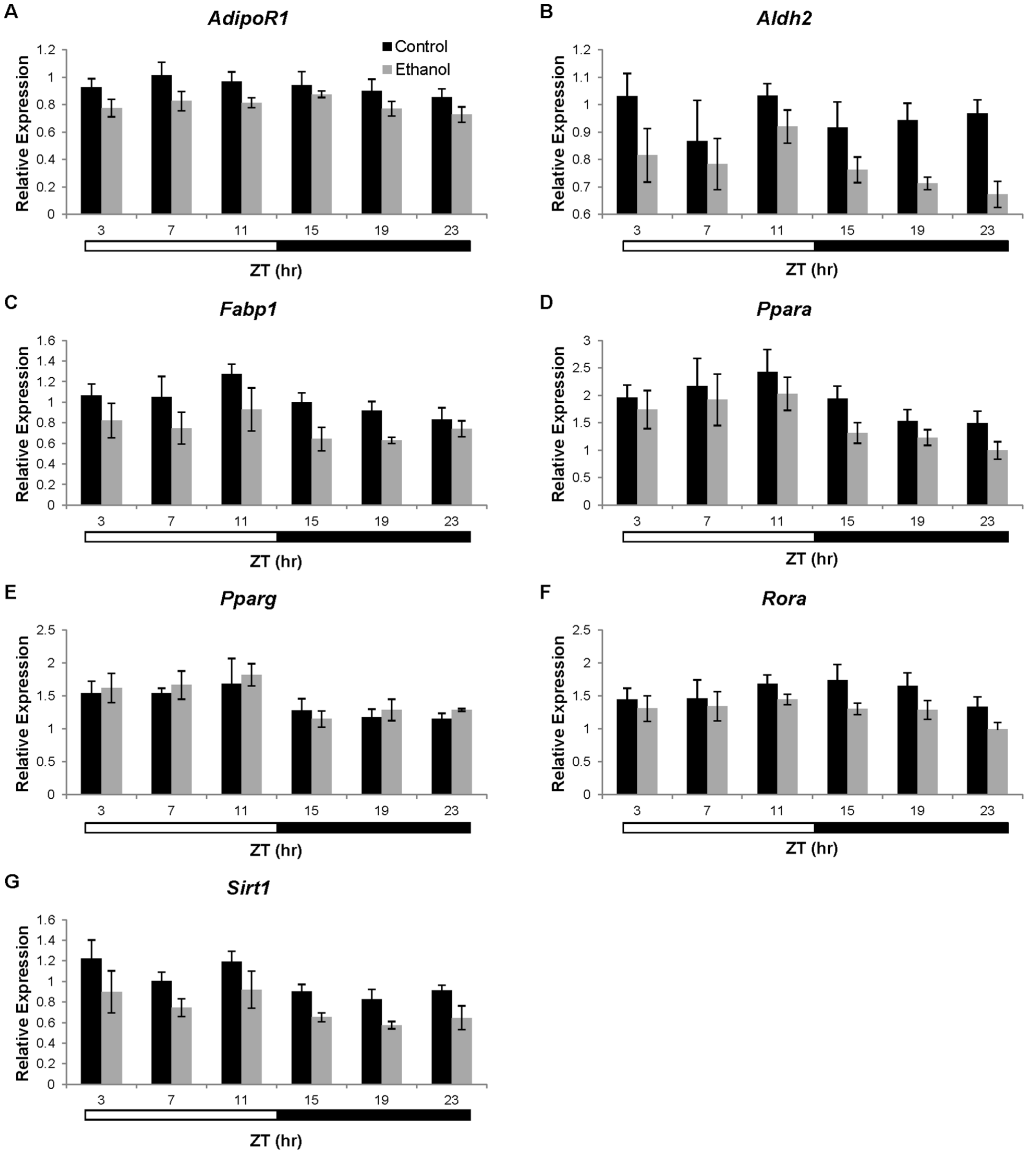
Expression of non-oscillating metabolism genes in the liver of C57BL/6J mice. Gene expression of A) *Adipor1*, B) *Aldh2*, C) *Fabp1*, D) *Ppara*, E) *Pparg*, F) *Rora*, and G) *Sirt1* was determined in liver from mice fed control (▪) or ethanol-containing (grey square) diets. Expression was normalized to the *Gapdh* housekeeping gene and is represented as fold-change compared to a single control mouse. Two-way ANOVA was used to determine significant effects of time and/or treatment on gene expression. Data are presented as mean ± SEM for n = 4–6 control and ethanol-fed mice at each time point.

To determine what effects clock disruption may have on metabolic genes within the liver, expression of genes involved in alcohol metabolism, glucose metabolism, lipid metabolism, NAD biosynthesis, and transcriptional regulation of these pathways (Table S1) was examined in the livers of wild-type C57BL/6J mice fed a control or ethanol-containing liquid diet. As described above, livers were harvested at 4-hr intervals over a 24-hr period. Genes demonstrating significant time effects according to two-way ANOVA were further examined using cosinor analysis. There were no significant main effects of time or ethanol-feeding on the expression of *Acadm*, *Adipor2*, *Dgat1*, *Fasn*, *G6pc*, *Gpam*, *Prkaa1*, *Rxra*, or *Smarcd1*, indicating that these genes are not under diurnal control in the liver. Further, there was no significant interaction effect on expression of these genes (Table S4). Expression of *Ppara*, *Pparg*, and *Sirt1* changed significantly with time (Table S4; Figure 5 d, e, and g), but these variations did not fit a cosine function (p>0.05; Table 3). Further, overall expression of *Ppara* and *Sirt1* was significantly lower in the ethanol-fed mice (Table S4; Figure 5 d and g). Ethanol-fed mice also had significantly lower levels of *Adipor1*, *Aldh2*, *Fabp1*, and *Rora,* but expression of these genes did not change with time (Table S4; Figure 5 a, b, c, and f).

**Table 3:** Cosinor analysis of metabolic genes in the liver.

Gene		r^2^	p-value	Amplitude	p-value	Acrophase	p-value	Mesor	p-value
*Acaca*	Control	0.109	0.2774	–	–	–	–	–	–
	Ethanol	0.288	0.0343	–		–		–	
*Adh1*	Control	0.368	0.0015	0.34	0.6284	14.04	0.9622	1.20	0.0295
	Ethanol	0.320	0.0202	0.29		13.98		1.38	
*Cpt1a*	Control	0.296	0.0084	–	–	–	–	–	–
	Ethanol	0.132	0.3060	–		–		–	
*Cyp2e1*	Control	0.234	0.0304	–	–	–	–	–	–
	Ethanol	0.081	0.5420	–		–		–	
*Dgat2*	Control	0.141	0.1654	–	–	–	–	–	–
	Ethanol	0.523	0.0003	–		–		–	
*Nampt*	Control	0.394	0.0008	1.06	0.3820	11.08	0.3889	1.70	0.2678
	Ethanol	0.535	0.0002	0.81		10.14		1.47	
*Pck1*	Control	0.255	0.0197	–	–	–	–	–	–
	Ethanol	0.163	0.2103	–		–		–	
*Pdk4*	Control	0.251	0.0245	–	–	–	–	–	–
	Ethanol	0.032	0.8444	–		–		–	
*Ppara*	Control	0.165	0.1093	–	–	–	–	–	–
	Ethanol	0.253	0.0590	–		–		–	
*Pparg*	Control	0.143	0.1615	–	–	–	–	–	–
	Ethanol	0.253	0.0600	–		–		–	
*Ppargc1a*	Control	0.232	0.0316	–	–	–	–	–	–
	Ethanol	0.218	0.1016	–		–		–	
*Ppargc1b*	Control	0.512	<0.0001	–	–	–	–	–	–
	Ethanol	0.208	0.1138	–		–		–	
*Sirt1*	Control	0.167	0.1057	–	–	–	–	–	–
	Ethanol	0.134	0.2991	–		–		–	
*Srebp1c*	Control	0.284	0.0111	–	–	–	–	–	–
	Ethanol	0.071	0.5978	–		–		–	

**Figure 6:**
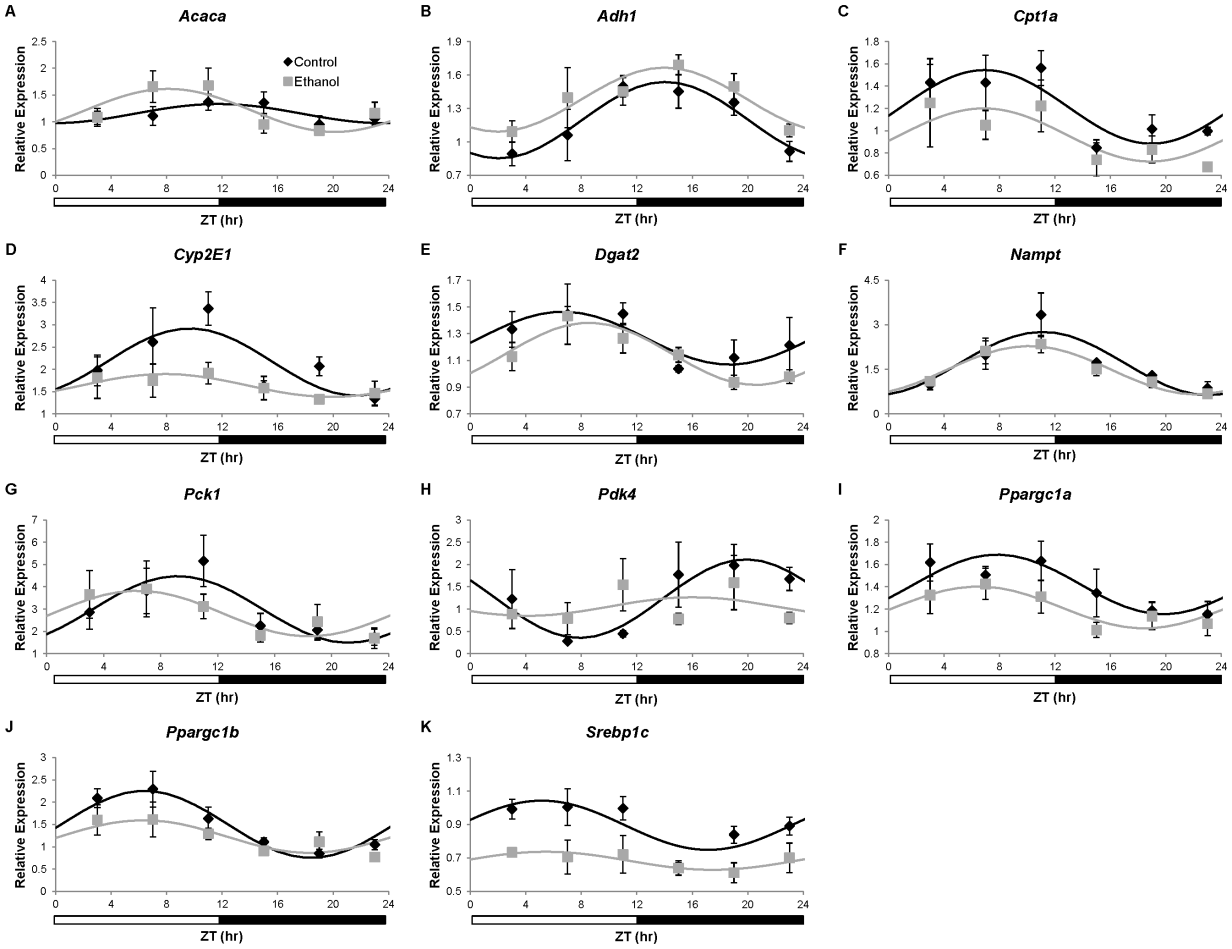
Expression of oscillating metabolic genes in the liver of wild-type C57BL/6J mice. Gene expression of A) *Acaca*, B) *Adh1*, C) *Cpt1a*, D) *Cyp2e1*, E) *Dgat2*, F) *Nampt*, G) *Pck1*, H) *Pdk4*, I) *Ppargc1a*, J) *Ppargc1a*, and K) *Srebp1c* in livers from mice fed control (♦) or ethanol-containing (grey square) liquid diets was determined using real-time quantitative PCR. Expression levels were normalized to the *Gapdh* housekeeping gene and are displayed as fold-change from a single control mouse. Cosinor analysis for curve fitting was performed using the nonlinear regression module in SPSS. Cosinor analysis results are included in Table 3. Data are presented as mean ± SEM for n = 4–6 control and ethanol-fed mice at each time point.

There were diurnal oscillations in the expression of *Adh1*, *Cpt1a*, *Cyp2e1*, *Nampt, Pck1*, *Pdk4, Ppargc1a*, *Ppargc1b*, and *Srebp1c* in livers from control mice (Table 3, Figure 6b, c, d, f, g, h, i, j, and k). Importantly, daily oscillations of *Cpt1a*, *Cyp2e1*, *Pck1*, *Pdk4*, *Ppargc1a*, *Ppargc1b*, and *Srebp1c* were lost in the livers of ethanol-fed mice. While diurnal oscillations of *Adh1* were maintained in the livers from ethanol-fed mice, the mesor was significantly increased by ethanol. Ethanol had no effect on the daily hepatic oscillations of *Nampt*. Interestingly, diurnal variations in the expression of *Acaca* and *Dgat2* were observed in ethanol-fed mice but not in control mice (Figure 6a and e). These studies demonstrate that ethanol alters the diurnal oscillations of metabolic genes within the liver, which may be a contributing factor to the development of alcoholic fatty liver.

## Discussion

In these studies, we utilized two complementary experimental models to test the hypothesis that chronic ethanol consumption alters the molecular circadian clock in the liver. Moreover, these systems allowed us to examine the phase relationship between the SCN and liver clock. First, we utilized wild-type C57BL/6J mice, which allowed us to examine expression of multiple clock and metabolic genes in the liver over a 24-hr period. Second, using the *Per2^Luc^* mouse model, we were able to examine the SCN-liver phase angle in a single mouse. Further, the *Per2^Luc^* mouse enabled us to verify that ethanol affects the cell autonomous clock in the liver and that the effects of ethanol are not solely acute, as they persist *ex vivo*. Collectively, our results show that chronic ethanol consumption disrupts the diurnal rhythms of clock genes in the livers of mice without significantly affecting the SCN. Daily oscillations of core clock genes (*Bmal1*, *Clock*, *Cry1*, *Cry2*, *Per1*, and *Per2*), as well as clock-controlled genes (*Dbp*, *Hlf*, *Nocturnin*, *Npas2*, *Rev-erbα*, and *Tef*), were altered in livers from C57BL/6J mice fed an ethanol-containing diet. In contrast, the SCN in these mice was largely unaffected. These observations were confirmed in the *Per2^Luc^* mouse experiment, which revealed a phase advance of PER2::LUC bioluminescence in liver explants from ethanol-fed mice with no effect on the SCN. Under control diet conditions, there was a 3–4 hr phase difference between the liver and SCN when measured as either *Per2* transcriptional expression or PER2::LUC bioluminescence, which is in agreement with previous reports [23]. However, in ethanol-fed mice, this phase relationship was reduced to 0.96 hr in *Per2^Luc^* mice and 1.73 hr in wild-type C57BL/6J mice. Independent of experimental model, our studies show that chronic ethanol consumption disrupts the hepatic clock without significantly affecting the SCN clock, demonstrating internal desynchrony between the liver and SCN clocks.

The relationship between alcohol consumption and peripheral clocks has been recently studied. Expression of core clock genes, including *hClock*, *hBmal1*, *hPer2*, *hCry1*, and *hCry2,* was decreased in peripheral blood mononuclear cells of alcohol-dependent male patients [29]. Similarly, the current study found a decrease in the mesor of all core clock genes (*Bmal1*, *Clock*, *Cry1*, *Cry2*, *Per1*, and *Per2*) in the liver of ethanol-fed mice. Further, neonatal alcohol exposure causes a phase advance of *Per2* liver expression in adult Sprague-Dawley rats [30], which was also observed in our studies of chronic alcohol exposure in adult mice. In contrast, exposure to 15% ethanol in drinking water had no effect on *Per2* or *Dbp* expression in the livers of wild-type or whole body *Clock* mutant mice, although steatosis was modestly increased in the mutant mice [20]. The higher fat content of the Lieber-DeCarli diet (35.9% fat) could be a contributing factor for these differences as most standard mouse chow diets contain much lower fat content (4% fat). Previously, we reported that the amount of ethanol-dependent triglyceride accumulation is dependent on the fat content of the diet suggesting an interaction between ethanol and fat [31]. However, daily oscillations of clock genes were shown to be maintained in the liver of mice fed a high-fat atherogenic diet (34.3% fat) for 5 wk, despite the presence of non-alcoholic steatohepatitis [32]. Studies using ethanol in drinking water typically show little to no hepatic steatosis with lower blood alcohol levels in the dark phase presumably due to metabolic tolerance [33], whereas in the current study blood alcohol levels in the dark phase (ZT15) were >300 mg/dL. Moreover, there are significant strain and sub-strain differences in sensitivity to ethanol-dependent hepatic steatosis [34] and differences between experimental model systems [35]. Finally, discrepancies could also be attributed to the expanded time-course in our study and/or different statistical analyses (cosinor analysis utilized in our study versus one-way ANOVA utilized in [20].

Alcohol consumption has also been shown to alter rhythms that are under neurobiological control. Shortened free-running periods of activity rhythm (a widely accepted measure of SCN function) have been noted in adult C57BL/6J mice provided 10% ethanol in drinking water [21]. Ethanol attenuates the responsiveness to brief photic stimulation in both rats and mice [21], [36], [37] and significantly alters expression of clock genes in the SCN of rats [30], [38]. Further, ethanol has been shown to inhibit glutamatergic (photic-like) and enhance serotonergic (non-photic) phase resetting in *ex vivo* brain slices [39]–[41]. However, these studies showed no changes in SCN phase in brain slices treated with ethanol alone, which is in agreement with the current study. Although we cannot comment on the activity rhythms of the mice used in the current study, we saw very few differences in the expression of core clock genes in the SCN between control and ethanol-fed mice, suggesting that ethanol consumption had no major effect on the central clock pacemaker. This disagreement with previous studies could be ascribed to differences in species (mice vs. rats), differences in ethanol feeding protocols, and duration of ethanol exposure [21], [30], [37], [38]. Further, it may be possible that any SCN effects induced by ethanol in the current study are masked by entrainment of the SCN to a 12∶12 hr light-dark cycle. For example, mice under a 12∶12 hr light-dark cycle provided ethanol in drinking water (free-choice or forced) show normal entrainment of their activity rhythms to the light-dark cycle, but aberrant entrainment to a weak skeleton photoperiod [36]. Importantly, future studies are needed to clarify whether ethanol affects intrinsic SCN clock gene activity.

While the molecular effects of ethanol on circadian clocks remain undefined, it is evident that ethanol alters the circadian system in the liver. It is well documented that the liver clock is a food-entrainable oscillator. In particular, phase shifting of the liver clock without SCN alterations has been observed in animals subjected to restricted feeding protocols (i.e., day-time feeding) [12], [13], [42] or high fat diet (45% energy from fat) [43]. Further, *ad libitum* high fat diet feeding also alters feeding patterns [44]. Therefore, it could be suggested that the liver-specific effects of ethanol on the molecular clock could be attributed to changes in feeding patterns. Although feeding patterns were not monitored in the current study, blood alcohol levels were elevated only during lights off (more active period), suggesting that ethanol-fed mice consumed the majority of their food during the dark period. It is possible that the observed alterations to the hepatic clock are directly related to ethanol metabolism, which largely occurs in the liver. In particular, ethanol is primarily metabolized by dehydrogenase enzymes (i.e., ADH and ALDH2), resulting in changes in NAD^+^ and NADH. Importantly, the DNA binding efficiency of the CLOCK-BMAL1 heterodimer to E-box elements is regulated by the redox state of NAD(H) cofactors [45], suggesting a possible link through which ethanol may alter hepatic clock gene expression.

Whether ethanol-induced clock disruption affects lipid metabolism in the liver and potentially contributes to alcoholic steatosis is not known. Genes encoding lipid metabolism enzymes demonstrate rhythmic expression in the liver [46]. As mentioned in the introduction, ethanol alters lipid homeostasis by decreasing PPARα activity and inducing SREBP1c activity, possibly through inhibition of SIRT1 and AMPK (reviewed in [5]). REV-ERBα, a nuclear orphan receptor that is also a clock-controlled gene, modulates the activity of SREBP1c in the liver [47]. Specifically, along with BMAL1 and REV-ERBβ, REV-ERBα binds to the *Srebp-1c* promoter [48], influencing its expression. REV-ERBα and REV-ERBβ are also essential for maintaining diurnal oscillations of *Pparα* expression in liver [48]. Moreover, the CLOCK-BMAL1 heterodimer directly activates expression of *Pparα* [49]. PER2, another core clock protein, interacts with the PPARα protein and influences the transcription of metabolic genes in liver [50]. PPARα is also known to directly activate the transcription of *Bmal1* in liver through the binding of PPAR response elements within the *Bmal1* promoter [51]. Also, SIRT1 influences circadian expression of *Bmal1*, *Per2*, and *Cry1*, and affects PER2 protein stability through deacetylation [52]. Taken together, these studies demonstrate cross-talk between the core components of the molecular clock and the same lipid metabolism enzymes that are influenced by ethanol consumption.

In our studies, ethanol decreased the mesor and amplitude of both *Bmal1* and *Rev-erbα* gene expression and phase advanced *Rev-erbα* in the liver. These changes in gene expression may have contributed to the abolished diurnal oscillation of *Srebp-1c*, decreased expression of *Pparα*, and an associated decrease in *Cpt1a* expression that were observed in the livers of ethanol-fed mice. We should also note that ethanol decreased the hepatic gene expression of *Srebp-1c*. Others have observed increased mature SREBP1c protein following administration of ethanol, with no difference in mRNA levels [4]. However, those experiments were performed at only one point within the circadian cycle.

Expression of *Sirt1* changed significantly with time and was decreased in ethanol-fed mice. It is possible that the decreased levels of *Sirt1* and *Pparα* expression could subsequently lead to down-regulation of clock genes, which was observed in the livers of ethanol-fed mice in this study. Ethanol also abolished the diurnal rhythms of *Ppargc1α* and *Ppargc1β* in the liver. PPARGC1α has been shown to induce expression of *Clock*, *Bmal1*, *Rev*-*erbα*, and *Rev*-*erbβ* and increase transcriptional activity of RORα [53], with deficiency of this protein contributing to hepatic steatosis after short-term starvation [54]. PPARGC1β knock-out mice have altered diurnal activity rhythms and develop hepatic steatosis after high-fat feeding [55]. We also observed that ethanol consumption induced diurnal oscillations in *Acaca* and *Dgat2* with gene expression peaking before the highest measured level of triglyceride in liver. It is possible that, in addition to the altered redox status of NAD(H), ethanol may disrupt the hepatic clock through changes in lipid metabolism enzyme activity, or vice versa. It still remains to be seen how the ethanol-induced alterations observed in the current study are associated with the development of alcoholic fatty liver disease. Further, the daily oscillations of alcohol metabolizing enzymes shown in this study suggest that the time-of-day of alcohol consumption may affect the toxicity associated with alcohol and this concept should be further explored.

### Conclusions

We have shown that ethanol affects diurnal rhythms of both clock and metabolism genes in the liver. Notably, we found that ethanol abolished diurnal oscillations of several key lipid metabolism genes. Further study is needed to determine the mechanisms by which these alterations in gene expression occur and whether they contribute to alcoholic fatty liver disease.

## Supporting Information

Figure S1 **Weekly body weights.** Body weight was monitored in A) C57BL/6J mice and B) Per2^Luc^ mice throughout the course of the ethanol-feeding protocol. Differences in body weight between control (♦) and ethanol-fed (▪) mice were determined using two-way repeated measures ANOVA. Data are presented as mean ± SEM for n = 33 control and ethanol C57BL/6J mice or n = 10 control and ethanol Per2^Luc^ mice.
(TIF)

Table S1 **Genes examined in suprachiasmatic nucleus and/or liver of C57BL/6 mice.**
(XLSX)

Table S2 **Two-way ANOVA results of clock gene expression in liver.**
(XLSX)

Table S3 **Two-way ANOVA results of gene expression in SCN.**
(XLSX)

Table S4 **Two-way ANOVA results of metabolic gene expression in liver.**
(XLSX)
