# Supplementary figures and images for: Chronic Ethanol Consumption Disrupts the Core Molecular Clock and Diurnal Rhythms of Metabolic Genes in the Liver without Affecting the Suprachiasmatic Nucleus
Source: PLoS One. 2013 Aug 12;8(8):e71684. doi: 10.1371/journal.pone.0071684 (PMC3741117; doi:10.1371/journal.pone.0071684)

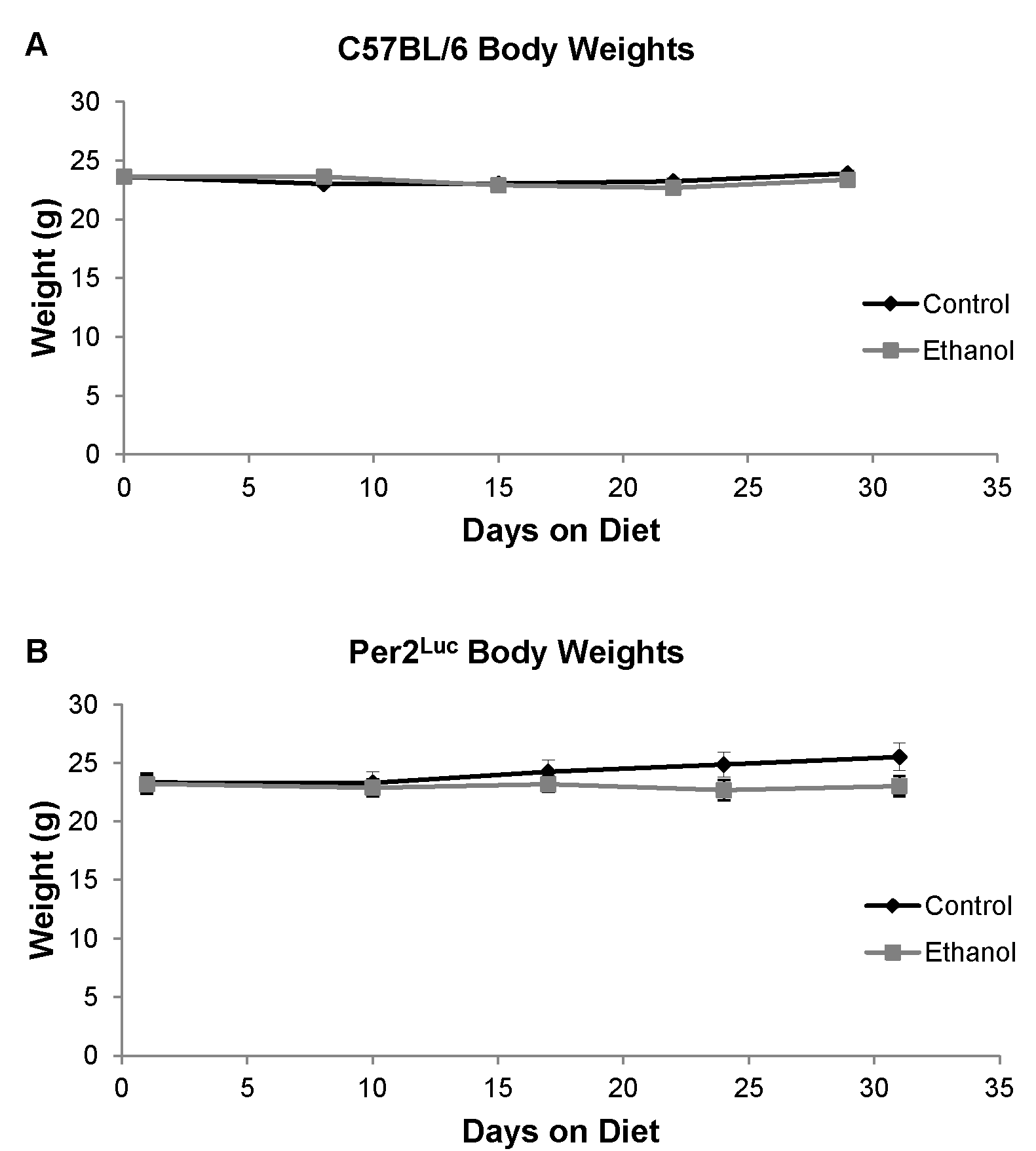

Supplement: Figure S1 — Weekly body weights. Body weight was monitored in A) C57BL/6J mice and B) Per2Luc mice throughout the course of the ethanol-feeding protocol. Differences in body weight between control (♦) and ethanol-fed (▪) mice were determined using two-way repeated measures ANOVA. Data are presented as mean ± SEM for n = 33 control and ethanol C57BL/6J mice or n = 10 control and ethanol Per2Luc mice. (TIF) [file pone.0071684.s001.tif]
